# Socio-ecological factors associated with probable depression among pregnant and parenting adolescent girls: findings from a cross-sectional study in Burkina Faso and Malawi

**DOI:** 10.1186/s12978-023-01588-x

**Published:** 2023-03-07

**Authors:** Anthony Idowu Ajayi, Elita Chamdimba, Nathalie Sawadogo, Nyawira Gitahi, Abdoul Moumini Tarnagda, Abdoul Kader Ilboudo, Alister Munthali, Chrissie Thakwalakwa, Emmanuel Oloche Otukpa, Boniface Ayanbekongshie Ushie, Caroline W. Kabiru

**Affiliations:** 1 Sexual, Reproductive, Maternal, New-born, Child and Adolescent Health (SRMNCAH) Unit, African Population and Health Research Center, Manga Close, Nairobi, Kenya; 2 Centre for Social Research, University of Malawi, P. O. Box 280, Zomba, Malawi; 3 Institut Supérieur des Sciences de La Population, Université Joseph Ki-Zerbo, B.P. 7118, Ouagadougou 03, Burkina Faso; 4 Dalla Lana School of Public Health, University of Toronto, 155 College St Room 500, Toronto, ON M5T 3M7 Canada

**Keywords:** Mental health, Depression, Pregnant and parenting adolescents, Adolescent mothers, Socio-ecological model

## Abstract

**Background:**

Pregnant and parenting adolescent girls are at risk of poor mental health because of stigma and social exclusion. Despite one in four girls starting childbearing by the age of 19 in Africa, no study, to the best of our knowledge, has examined the multi-layered factors (individual, family, friends, and neighborhood-related factors) associated with depressive symptoms among pregnant and parenting girls in Africa. Our study contributes to addressing this gap by examining the socio-ecological factors associated with depression symptoms among pregnant and parenting adolescent girls.

**Methods:**

Our study adopted a cross-sectional design. Between March and September 2021, we interviewed 980 pregnant and parenting adolescent girls in Ouagadougou, Burkina Faso, and 669 in Blantyre, Malawi. We recruited pregnant and parenting adolescent girls in randomly selected urban and rural enumeration areas in Burkina Faso (n = 71) and Malawi (n = 66). We assessed depressive symptoms using the Patient Health Questionnaire (PHQ-9), which generated an overall score of 27. We considered a score of 10 or more as probable depression. We also obtained information on individual, family, friends, and neighborhood characteristics. We employed logistic regression models to examine the significant factors associated with probable depression among pregnant and parenting adolescent girls.

**Results:**

The prevalence of probable depression was 18.8% and 14.5% in Burkina Faso and Malawi, respectively. At the individual level, having secondary education was significantly associated with a lower likelihood of probable depression in Malawi (AOR: 0.47; 95% CI 0.27–0.82) but not in Burkina Faso. At the family level, denying paternity (AOR: 3.14; 95% CI 1.34–7.11 in Malawi) and no parental support (AOR: 2.08; 95% CI 1.22–3.55 in Burkina Faso) were associated with higher odds of probable depression. At the community level, perceived neighborhood safety was associated with a lower likelihood of probable depression in Malawi (AOR: 0.74; 95% CI 0.61–0.89) and Burkina Faso (AOR: 0.81; 95% CI 0.73–0.90). Having a safety net within the community was associated with lower odds of probable depression in Burkina Faso (AOR: 0.87; 95% CI 0.78–0.96) but not in Malawi.

**Conclusion:**

Depressive symptoms are common among pregnant and parenting adolescents, suggesting the need to screen them regularly for depression during antenatal and postnatal visits. Factors associated with depression among pregnant and parenting girls operate at multiple levels suggesting a need for multilevel interventions that address all areas of vulnerabilities.

## Abstract (highlights)

Pregnant and parenting adolescents are at a higher risk of poor mental health. If unaddressed or untreated, the consequences may be dire throughout an individual’s life. About one in four girls start childbearing before their 19th birthday in Africa. Most of these pregnancies are unintended or unplanned and occur outside wedlock, bringing untoward disruption to every facet of the girl’s life. The burden of early parenthood and the stigma of starting childbearing too early and outside wedlock can affect the mental wellness of girls. Yet only a few studies have focused on understanding the burden and drivers of poor mental health among pregnant and parenting girls. Our study contributes to the research on depression among pregnant and parenting girls, highlighting its burden and individual, family, and community drivers. We conducted this study in Ouagadougou, Burkina Faso, and Blantyre, Malawi, between March and September 2021. A total of 980 and 669 pregnant and parenting girls were recruited into this study in Burkina and Malawi. Our analysis shows that 18.8% and 14.5% of pregnant and parenting girls in Burkina Faso and Malawi, respectively, had probable depression. Depression symptoms was higher when girls were older than 17 years, suffered violence from their partners, their partners denied paternity, received no support from their partners and parents, lived in violent neighborhoods, and had no support system in the community. We conclude that depression is high among pregnant and parenting girls. It necessitates screening them for depression during antenatal and postnatal care visits. To effectively address depression among these girls, interventions must canvass support at the family and community levels.

## Introduction

Mental health problems significantly contribute to the disease burden among adolescents and young adults globally [1]. Half of all mental health conditions begin in adolescence, and one in six people aged 10–19 years suffer from some form of mental disorder [2]. Depression and suicide are among the leading causes of death and disabilities among adolescents [1]. However, most mental conditions are undiagnosed and untreated, especially in resource-poor settings [3]. Untreated mental conditions have detrimental consequences across the life course, damaging physical health and overall wellness and robbing individuals of opportunities to lead fulfilling lives as adults [3]. Untreated mental health problems among adolescents are also linked to low educational achievement, unemployment, substance abuse, risky behaviors, crime, and self-harm, leading to a heightened lifetime risk of morbidity and mortality [2].

The reasons why adolescents are susceptible to poor mental health outcomes are multifaceted. Childhood abuse, family, school, and neighborhood violence, poverty, loss and grief, social exclusion, early unintended pregnancy, and chronic diseases are among the risk factors for poor mental health outcomes among adolescents [3–6]. Vulnerable adolescents, including those pregnant, living with HIV or disability, orphaned, trafficked, and forced into sex work, are disproportionately susceptible to poor mental health, including depression, anxiety, suicide ideation, self-harm, and traumatic stress[7]. There is evidence that pregnant and parenting adolescent (PPA) girls are disproportionately exposed to physical abuse, more stressful life events, and postpartum depressive symptoms than adult women [4, 8, 9]. For example, the risk of postpartum depression is doubled in adolescent mothers compared to their non-parenting peers and adult women [10]. Elevated levels of mental health challenges among PPA girls are a function of their age and other social disadvantages and adversities that precede pregnancy [5, 8].

Some of the disadvantages that make adolescent girls vulnerable to early and unintended pregnancies also elevate their risk of poor mental health. Studies from the US show that the childhoods of adolescent mothers from resource-poor communities and poor households are characterized by social inequality, chronic stress, violence, food insecurity, and poverty, all of which increase their risk of experiencing rape and sexual violence and unintended pregnancy. Some became pregnant due to sexual abuse, a risk factor for poor mental health [5]. When they become mothers, their adversities become compounded by the stress of parenting, poverty, lack of resources to care for their babies, and the stigma of early childbearing outside wedlock, especially when the sexual partners deny responsibility and offer no support [11, 12].

Much of the literature on adolescent mental health is concentrated in the Global North [11, 12]. More recently, there has been an increased focus on the mental health of adolescents living with HIV in Africa [13–17]. However, limited research, program interventions, and policies have addressed pregnant and parenting adolescents' mental health and wellness. Even though Africa has the highest prevalence of adolescent childbearing globally, interventions to improve the mental health and wellbeing of adolescent mothers are concentrated in the global north [18]. Diagnosing and treating mental distress in many African countries is limited and under-resourced. Most people suffering from poor mental health lack access to much-needed care. While a few studies have examined the factors associated with depression symptoms among PPA girls in sub-Saharan Africa [19, 20], none to the best of our knowledge has comprehensively examined the role of individual, family, friends, and neighborhood-related factors associated with depressive symptoms among pregnant and parenting girls. Our study addresses this gap by examining the socio-ecological factors associated with depression symptoms among PPA girls in rural and urban areas in two districts of Burkina Faso and Malawi.

## Theoretical underpinning

This study drew from the socio-ecological model to understand the multi-layered factors associated with probable depression among pregnant and parenting adolescents [21–24]. We argue that factors predisposing PPA girls to probable depression are complex and multi-layered. The interplay of individual, family, friend, and community level factors combine to expose girls to poor or good mental health [22, 24]. When girls become pregnant, often outside wedlock, they face social exclusion at home, from friends and in the community, which could culminate in poor mental health [9]. Early and unintended pregnancy constitutes a barrier to girls’ education and economic empowerment [25]. It exposes them to physical and emotional abuse from their families, partners, and community [26]. Often, their friends judge, mock, isolate, and gossip about them, which could lead to poor mental health [20]. Because they are very young mothers, they need much support to navigate parenting, support that is often not available to them [27]. Sometimes, their partners deny paternity and refuse to support them, which make them vulnerable to poor mental health [28, 29]. Previous studies have also shown that community or neighborhood-level factors are relevant in understanding poor mental health among pregnant and parenting adolescents [30–32]. Girls who live in poor neighborhoods where crime and violence are rampant have poorer mental health than their counterparts [31, 32]. Without support from family, partners, friends, and community, adolescent mothers may be unable to deal with the compounded challenges of motherhood.

## Methods

### Study design and setting

We analyzed data from a cross-sectional study that examined pregnant and parenting adolescents’ lived experiences in Burkina Faso in West Africa and Malawi in Southern Africa. We selected these countries purposively because they have some of the highest adolescent childbearing rates in the world—136 births per 1000 girls in Malawi and 132 births per 1000 girls in Burkina Faso [33]. The choice of Blantyre and Ouagadougou was convenient given the data was collected during COVID-19 and adolescent childbearing is common in all regions of the countries.

### Sampling procedure

We used a two-stage probability sampling approach to recruit pregnant and parenting girls into the study. The first stage involved random sampling of urban and rural enumeration areas (EAs). Overall, 66 EAs (rural = 26 and urban = 40) were sampled in Blantyre, Malawi, and 71 EAs (rural = 29 and urban = 42) were selected in the Central region that includes Ouagadougou and six rural municipalities (Pabré, Loumbila, Tanghin Dassouri, Saaba, Komsilga and Koubri) around the town in Burkina Faso. In the second stage, we conducted a household listing in the sampled EAs to identify households with eligible pregnant and parenting adolescent girls. To be eligible to participate, girls had to be aged 10–19 years, ever been pregnant, pregnant or have a biological child, and be mentally capable of responding to the questions in English, French, Chichewa, or the local languages. All identified adolescents were selected per household. In total, we successfully interviewed 980 and 669 adolescent girls in Ouagadougou and Blantyre, giving a response rate of 96.2% (n = 980/1019) and 98.5% (n = 669/679), respectively.

### Data collection procedures

Data were collected using a structured questionnaire adapted from existing instruments, such as the Global Early Adolescent Study tools [34], and Patient Health Questionnaire-9 (PHQ-9) [35–37]. We administered the study questionnaires in the English, French and national languages: Chichewa in Malawi and Moore and Dioula in Burkina Faso. The questionnaire was piloted in both study settings, and identified issues were corrected before we administered the final version. Data collection took place between March and September 2021. Trained enumerators used Android-powered tablets and smartphones with SurveyCTO to collect and upload data to a secured server. The research teams were managed by supervisors who monitored the data collection process by cross-checking for errors daily before syncing the data onto the server.

### Main outcome

We used the Patient Health Questionnaire-9 (PHQ-9) to determine depression symptoms among PPA girls [35–37]. The PHQ-9 has over 75% sensitivity and specificity for screening depression in adolescents [38] and is widely used and validated in African settings [39–42]. The PHQ-9 is a nine-item self-assessment instrument with response options of 0 (not at all); 1 (several days), 2 (more than half the days), and 3 (nearly every day). We asked each respondent how often they have been bothered by the following problems over the last two weeks: having little interest or pleasure in doing things; feeling down or hopeless; having insomnia or oversleeping; fatigue; loss of appetite or over-eating, having low self-esteem; trouble concentrating; restlessness or slowness; and negative self-thoughts, including self-harm. Scores of 10 to 27 are further grouped as probable depression and zero to nine are grouped as no probable depression. The questions have a high degree of internal consistency in our study with a Cronbach's alpha coefficient of 0.84 and 0.85 in Malawi and Burkina Faso.

### Covariates

We included three levels of covariates, individual, family and friends, and community levels. At the individual level, we included age (13–16, 17, 18, and 19 years), marital status (married/cohabiting, separated/divorced, and single), education level (no education/primary and secondary), marital status at the time of first pregnancy (single, engaged and married), personal reaction to the pregnancy (scared, happy, disappointed), and birth status (ever given birth/has at least one child and still pregnant/never given birth). At the family and friend level, we included exposure to intimate partner violence, parent’s reactions to pregnancy (happy upset and neutral), friends’ reactions to pregnancy (happy upset and neutral), partner’s reactions to pregnancy (happy, denied, upset and neutral), parental support (good, fair, poor/no support), friend’s support (good, fair, poor/no support), and partner’s support (good, fair, poor/no support). Exposure to intimate partner violence (IPV) was generated using the Demographic and Health Survey IPV module and any experience of sexual, emotional and physical violence was grouped as “Yes” and no experience was grouped as “No”.

At the community level, we included perceived neighborhood safety and social assets and safety nets. Seven questions were used to assess neighborhood safety, and these questions have a moderate internal consistency and validity with an alpha coefficient of 0.42. Each participant responded by indicating the degree of agreement or disagreement to questions about feeling safe walking around their community during the day and after dark, feeling scared of being raped, and having previously been robbed or indecently touched. Social capital was measured using a scale comprising seven items (alpha coefficient of 0.66). The questions explored if PPA girls agree or disagree with statements on having someone they can rely on for child support, advice, loan, psychosocial support, or help.

### Statistical analysis

We used SPSS (version 25) for data cleaning and Stata (version 15 for Windows) [43] for all the statistical analyses. The survey datasets were weighted to correct for over or under sampling of some areas. Descriptive statistics were produced using means, standard deviations, frequencies, and percentages to present the demographic characteristics of the respondents and observations of probable depression and no depression. We fitted adjusted and unadjusted models to examine individual, family/friend, and community levels correlate of probable depression among pregnant and parenting girls. A p-value < 0.05 was considered statistically significant, and 95% confidence intervals were estimated. Model 1 is the unadjusted model showing the effect of each variable on the study outcome, and Model 2 is the adjusted model showing the effect of each variable after controlling for covariates. We dropped living with both parents and orphanhood status from the multivariable analysis because of multicollinearity. Living with both parents and orphanhood status significantly correlate with parental support.

### Ethical considerations

The Research Ethics Committee of the University of Malawi (UNIMAREC) and Burkina Faso’s Ministry of Health Ethics Committee for Health Research approved the study protocol. Interviews were conducted in private spaces, and participants' rights to confidentiality and anonymity were respected during and after data collection. Married pregnant and parenting adolescents provided oral and written informed consent after the research assistants explained the aim of the study and the use of data to them. Parents and guardians of single pregnant girls gave informed consent, and their wards assented to the study. We observed all IRB’s ethics guidelines for research on human subjects.

## Results

### Descriptive findings

The average age of pregnant and parenting girls in Malawi and Burkina Faso was 18.9 ± 1.2 years and 18.4 ± 0.9 years, respectively. Table 1 presents the weighted individual, family, and community characteristics of respondents. In both Malawi (66.9%) and Burkina Faso (80.6%), most PPA girls were aged 18 to 19. More respondents had ever worked for pay in Burkina Faso (36.9%) compared to Malawi (28.7%). Similarly, more respondents in Burkina Faso (84.2%) than in Malawi (51.0%) were not living with both parents. While over three-quarters of PPA girls in Burkina Faso (75.6%) were married/cohabiting, less than half in Malawi (45.8%) were married. In contrast, a higher proportion of PPA girls in Malawi (63.3%) described parental support as good compared to those from Burkina Faso (47.2%). However, more PPA girls from Burkina Faso (75.2%) described the partners/spousal support as good compared to those from Malawi (61.9%). More respondents got pregnant outside of formal union in Malawi (80.6%) than in Burkina Faso (56.4%).

**Table 1:** Weighted individual, family and friend, and community characteristics of respondents

Variables	Malawi		Burkina Faso	
	Weighted sample		Weighted sample	
	Frequency	Percent	Frequency	Percent
Residence				
Rural	275	38.2	398	19.1
Urban	394	61.8	582	80.9
Age				
13–16	87	13.3	71	6.9
17	133	19.9	127	12.5
18	199	29.8	313	32.1
19	249	37.1	469	48.5
Education level				
Primary or less	440	65.4	526	53.0
Secondary	229	34.6	454	47.0
Ever worked for pay				
Yes	191	28.7	344	36.9
No	478	71.3	636	63.1
Orphanhood status				
Double orphan	39	5.8	54	6.1
Single orphan	181	27.1	208	20.7
Non-orphan	449	67.1	718	73.2
Living with both parents				
Not living with both parents	341	50.9	832	84.2
Living with one parent	176	26.3	63	6.8
Living with both parents	152	22.8	85	9.1
Marital status				
Married	307	45.8	761	75.6
Separated	79	11.8	59	59
Single	283	42.3	160	17.8
Birth status				
Ever given birth/has at least one child	144	21.4	297	28.8
Still pregnant/never gave birth	525	78.6	686	71.2
Personal reaction to pregnancy				
Scared	183	27.3	335	36.5
Happy	187	27.8	435	42.5
Disappointed	299	44.9	210	21.0
Marital status at the time of pregnancy				
Single	539	80.7	537	56.4
Engaged	15	2.3	38	3.5
Married	115	17.0	405	40.2
Parental support				
Good	425	63.3	445	47.2
Fair	136	20.5	371	37.0
Poor	33	4.9	75	7.1
No support	75	11.2	89	8.7
Friends support				
Good	155	22.9	250	25.0
Fair	137	21.0	433	45.8
Poor	83	12.4	61	6.4
No support	294	43.7	236	22.8
Partner support				
Good	415	61.9	741	75.2
Fair	108	16.4	142	14.1
Poor	48	7.2	96	10.7
No support	98	14.5	1	0.05
Neighborhood safety (median)	669	6	980	5
Social assets and safety net (median)	669	4	980	5

^*^Median computed for “neighborhood safety” and “social assets and safety net”

### Probable depression prevalence

Table 2 presents the prevalence of probable depression by respondents’ individual, family, and community-level characteristics. The prevalence of probable depression was 14.5% and 18.8% in Malawi and Burkina Faso, respectively. The prevalence of probable depression was higher among single PPA girls compared to married adolescents in both Malawi (single 17.7% vs. married 9.8%) and Burkina Faso (single (25.4% vs. married 16.6%). Probable depression prevalence was lower among those who received good support from parents, friends, and partners than those who received poor or no support in both countries (Table 2). Similarly, probable depression prevalence was lower among respondents who were married before getting pregnant than those single when they got pregnant in both study sites. Probable depression prevalence was higher among respondents who reported intimate partner violence than those who did not in both study sites.

**Table 2:** Depression symptoms prevalence by individual, family, and community-level characteristics

Variables	Malawi		Burkina Faso	
	Not depressed % (CI)	Probably depressed % (CI)	Not depressed % (CI)	Probably depressed % (CI)
All	85.5 (82.5–87.9)	14.5 (12.1–17.5)	81.2 (78.3–83.7)	18.8 (16.3–21.7)
Age				
13–16	86.5 (77.9–92.1)	13.5 (7.9–22.1)	77.6 (65.0–86.6)	22.4 (13.4–35.0)
17	84.9 (77.8–90.0)	15.1 (10.0–22.2)	70.8 (61.3–78.9)	29.2 (21.1–38.7)
18	84.6 (78.9–88.9)	15.4 (11.1–21.1)	82.6 (77.5–86.7)	17.4 (13.3–22.5)
19	86.1 (81.1–89.9)	13.9 (10.1–18.9)	83.4 (79.3–86.8)	16.6 (13.2–20.7)
Education level				
Primary or less	83.3 (79.4–86.5)	16.7 (13.5–20.6)	82.6 (78.7–86.0)	17.4 (14.0–21.3)
Secondary	89.6 (85.0–92.9)	10.4 (7.1–15.0)	79.6 (75.2–83.3)	20.4 (16.7–24.8)
Marital status				
Married	90.2 (86.3–93.0)	9.8 (7.0–13.7)	83.4 (80.3–86.1)	16.6 (13.9–19.7)
Separated	78.5 (68.1–86.2)	21.5 (13.8–31.9)	73.4 (59.9–83.6)	26.6 (16.4–40.1)
Single	82.3 (77.4–86.3)	17.7 (13.7–22.6)	74.6 (66.7–81.1)	25.4 (18.9–33.3)
Ever worked for pay				
Yes	80.4 (74.2–85.4)	19.6 (14.6–25.8)	78.3 (73.2–83.7)	21.7 (17.3–26.8)
No	87.5 (84.1–90.3)	12.5 (9.7–15.9)	82.8 (79.4–85.8)	17.2 (14.2–20.6)
Orphanhood status				
Double orphan	79.5 (64.0–89.5)	20.5 (10.5–36.0)	73.1 (58.6–83.9)	26.9 (16.1–41.4)
Single orphan	81.4 (75.0–86.4)	18.6 (13.6–25.0)	78.0 (71.0–83.7)	22.0 (16.3–29.0)
Non-orphan	87.6 (84.2–90.4)	12.4 (9.6–15.8)	82.7 (79.5–85.6)	17.3 (14.4–20.5)
Living with both parents				
Not living with both parents	85.7 (81.7–88.9)	14.3 (11.1–18.3)	81.5 (78.4–84.3)	18.5 (15.7–21.6)
Living with one parent	83.8 (77.7–88.4)	16.2 (11.6–22.3)	76.6 (63.4–86.1)	23.4 (13.9–36.6)
Living with both parents	86.9 (80.7–91.3)	13.1 (8.7–19.3)	81.5 (71.0–88.8)	18.5 (11.2–29.0)
Residence				
Rural	83.3 (78.3–87.3)	16.7 (12.7–21.7)	84.4 (80.5–87.7)	15.6 (12.3–19.5)
Urban	86.8 (83.1–89.8)	13.2 (10.2–16.9)	80.4 (77.0–83.4)	19.6 (16.6–23.0)
Parental support				
Good	89.0 (85.6–91.6)	11.0 (8.4–14.4)	85.3 (82.2–87.9)	14.7 (12.1–17.8)
Fair	77.5 (69.7–83.8)	22.5 (16.2–30.3)	72.8 (64.1–80.1)	27.2 (19.9–35.9)
Poor	82.4 (65.8–91.9)	17.6 (8.1–34.2)	63.5 (52.7–73.1)	36.5 (26.9–47.3)
No support	81.6 (71.4–88.7)	18.4 (11.3–28.6)	100.0	0.0
Friends support				
Good	91.7 (86.2–95.1)	8.3 (4.9–13.8)	89.0 (83.9–92.6)	11.0 (7.4–16.1)
Fair	82.7 (75.4–88.1)	17.3 (11.9–24.6)	82.6 (78.4–86.2)	17.4 (13.8–21.6)
Poor	85.4 (76.0–91.6)	14.6 (8.4- 24.0)	60.6 (46.8–72.9)	39.4 (27.1–53.2)
No support	83.5 (78.9–87.3)	16.5 (12.7–21.1)	75.5 (68.8–81.1)	24.5 (18.9–31.2)
Partner support				
Good	89.6 (86.1–92.2)	10.4 (7.8–13.9)	86.7 (82.8–89.8)	13.3 (10.2–17.2)
Fair	78.9 (70.5–85.4)	21.1 (14.6–29.5)	80.4 (75.4–84.5)	19.6 (15.5–24.6)
Poor	75.7 (61.2–86.0)	24.3 (14.0–38.8)	61.5 (48.9–72.8)	38.5 (27.2–51.1)
No support	80.2 (71.3–86.8)	19.8 (13.2–28.7)	70.7 (59.1–80.1)	29.3 (19.9–40.9)
Belonging to a social group				
Yes	83.9 (79.0–87.8)	16.1 (12.2–21.0)	81.0 (76.7–84.6)	19.0 (15.4–23.3)
No	86.5 (82.8–89.5)	13.5 (10.5–17.2)	81.4 (77.4–84.8)	18.6 (15.2–22.6)
Marital status at the time of pregnancy				
Single	83.9 (80.5–86.7)	16.1 (13.3–19.5)	76.0 (71.9–79.8)	24.0 (20.2–28.1)
Engaged	93.1 (63.7–99.0)	6.9 (1.0–36.3)	79.8 (61.5–90.7)	20.2 (9.3–38.5)
Married	92.0 (85.3–95.8)	8.0 (4.2–14.7)	88.5 (84.6–91.5)	11.5 (8.5–15.4)
Personal reaction to pregnancy				
Scared	84.4 (78.3–89.0)	15.6 (11.0–21.7)	77.9 (72.7–82.4)	22.1 (17.6–27.3)
Happy	90.8 (85.7–94.2)	9.2 (5.8–14.3)	87.3 (83.3–90.4)	12.7 (9.6–16.7)
Disappointed	82.8 (78.1–86.8)	17.2 (13.3–21.9)	74.5 (67.4–80.4)	25.5 (19.6–32.6)
Birth status				
Ever given birth/has at least one child	84.7 (77.7–89.8)	15.3 (10.2–22.3)	73.9 (68.0–79.1)	26.1 (20.9–32.0)
Still pregnant/never gave birth	85.7 (82.3–88.4)	14.3 (11.6–17.7)	84.1 (80.9–86.9)	15.9 (13.1–19.1)
Exposed to IPV				
Yes	73.2 (70.8–80.9)	23.8 (19.1–29.2)	77.9 (74.4–81.0)	22.1 (19.0–25.6)
No	91.6 (88.4–93.9)	8.4 (6.1–11.6)	91.4 (86.6–94.7)	8.6 (5.3–13.4)
Neighborhood safety	5.40(5.30–5.49)	4.86(4.63–5.09)	4.73(4.61–4.84)	4.15(3.91–4.40)
Social assets and safety nets	4.10(3.94- 4.26)	4.01(3.63–4.39)	4.85(4.72–4.98)	4.32(4.04–4.59)

^*^Mean differences were computed for “neighborhood safety” and “social assets and safety net

### Multivariable findings

Our models examined individual, family and friend, and community-level factors associated with probable depression among pregnant and parenting adolescent girls. The individual-level factors associated with probable depression in the unadjusted model were age, marital status, educational level, and birth status (Table 3). After adjusting for covariates, increasing age was associated with higher odds of probable depression in both Malawi and Burkina Faso. However, the effect size reached a statistically significant level only in Malawi. While being single and separated was significantly associated with a higher likelihood of probable depression in the unadjusted model, the effect size did not reach a significant level after controlling for relevant covariates. Having secondary education was significantly associated with a lower likelihood of probable depression in Malawi but not in Burkina Faso. Parenting girls were more likely to report lower levels of probable depression compared to those currently pregnant in Burkina Faso but not in Malawi.

**Table 3:** Multivariable logistic regression models showing individual, family and friend, and community-level factors associated with depression symptoms among pregnant and parenting girls

Variables	Malawi		Burkina Faso	
	Model 1	Model 2	Model 1	Model 2
Age				
13–16	1	1	1	1
17	1.12 (0.52–2.43)	2.22 (0.94–5.21)	1.37 (0.68–2.73)	1.86 (0.87–4.02)
18	1.17 (0.57–2.40)	2.84 (1.24–6.49)*	0.78 (0.41–1.48)	1.14 (0.55–2.37)
19	1.04 (0.51–2.10)	3.02 (1.33–6.87)**	0.69 (0.37–1.28)	1.24 (0.61–2.54)
Marital status				
Married	1	1	1	1
Separated	2.53 (1.31–4.88)**	1.62 (0.77–3.41)	2.01 (1.09–3.68)*	1.08 (0.48–2.42)
Single	2.03 (1.25–3.29)**	1.73 (0.89–3.36)	1.86 (1.24–2.79)**	0.76 (0.43–1.34)
Highest level of education				
Primary or less	1	1	1	1
Secondary	0.58 (0.35–0.95)*	0.47 (0.27–0.82)**	1.39 (1.004–1.93)*	1.34 (0.91–2.02)
Marital status at the time of pregnancy				
Single	1	1	1	
Engaged	0.37 (0.05–2.82)	0.43 (0.05–3.32)	0.75 (0.32–1.75)	1.40 (0.51–3.83)
Married	0.44 (0.21–0.89)*	0.76 (0.29–1.98)	0.42 (0.29–0.60)***	0.65 (0.30–1.43)
Personal reaction to pregnancy				
Scared	1	1	1	1
Happy	0.53 (0.28–1.01)	0.89 (0.39–2.04)	0.51 (0.34–0.75)***	1.01 (0.54–1.89)
Disappointed	1.12 (0.68–1.84)	1.00 (0.58–1.73)	1.26 (0.84–1.89)	0.79 (0.49–1.26)
Birth status				
No birth/still pregnant	1	1	1	1
Ever birthed/has at least one child	0.94 (0.56–1.57)	0.60 (0.33–1.10)	0.55 (0.39–0.76)***	0.65 (0.44–0.94)*
Exposed to IPV				
No	1	1	1	1
Yes	3.44 (2.19–5.39)***	2.87 (1.77–4.66)***	2.72 (1.64–4.49)***	2.17 (1.23–3.82)**
Parent's reaction				
Happy	1	1	1	1
Upset	2.60 (1.30–5.20)**	1.38 (0.45–4.21)	2.30 (1.59–3.32)***	0.67 (0.29–1.57)
Neutral	1.95 (0.86–4.45)	0.84 (0.27–2.57)	2.47 (1.42–4.29)**	0.88 (0.37–2.07)
Friend's reaction				
Happy	1	1	1	1
Upset	2.15 (1.01–4.58)*	1.12 (0.40–3.17)	4.34 (2.55–7.38)***	1.99 (0.99–3.99)
Neutral	1.83 (0.90–3.72)	0.90 (0.34–2.37)	2.12 (1.44–3.09)***	1.09 (0.64–1.85)
Partner's reaction				
Happy	1	1	1	1
Denied	2.13 (1.14–4.00)*	1.09 (0.44–2.72)	5.22 (3.18–8.57)***	3.14 (1.34–7.11)**
Upset	0.99 (0.51–1.93)	0.58 (0.26–1.27)	2.76 (1.75–4.35)***	1.52 (0.83–2.79)
Neutral	1.45 (0.85–2.48)	1.16 (0.60–2.25)	1.31 (0.72–2.38)	0.78 (0.38–1.59)
Parental support				
Good	1	1	1	1
Fair	2.37 (1.44–3.93)***	1.95 (1.08–3.52)*	1.47 (1.002–2.17)*	1.19 (0.75–1.89)
No/Poor	1.83 (1.03–3.24)*	1.59 (0.82–3.10)	3.25 (2.12–4.99)***	2.08 (1.22–3.55)**
Friends support				
Good	1	1	1	1
Fair	2.32 (1.13–4.76)*	1.36 (0.61–3.07)	1.62 (1.01–2.60)*	1.04 (0.61–1.77)
No/poor	2.11 (1.12–3.96)*	1.17 (0.54–2.55)	2.94 (1.83–4.74)***	1.49 (0.85–2.64)
Partner support				
Good	1	1	1	1
Fair	2.34 (1.34–4.09)**	1.23 (0.64–2.37)	2.46 (1.61–3.74)***	1.78 (1.02–3.09)*
No/poor	2.43 (1.47–4.02)***	1.37 (0.68–2.76)	3.38 (2.12–5.39)***	1.21 (0.58–2.52)
Neighborhood safety	0.71 (0.60–0.83)***	0.74 (0.61–0.89)**	0.82 (0.75–0.90)***	0.81 (0.73–0.90)***
Social assets and safety nets	0.98 (0.88–1.07)	0.94 (0.82–1.06)	0.87 (0.80–0.94)***	0.87 (0.78–0.96)**

Exponentiated coefficients; 95% confidence intervals in brackets *p < 0.05, **p < 0.01, ***p < 0.001

The family and friend-related factors significantly associated with probable depression in the unadjusted model were IPV, parental, partner, and friends' reactions to pregnancy, and their level of support. However, only exposure to IPV, denial of paternity, and parental and partner support reached a significant level in the adjusted model. Pregnant and parenting girls who reported experiencing IPV were more likely to report depression symptoms than those who did not in both study settings. In Malawi and Burkina Faso, PPA girls who reported that their partner denied paternity were more likely to report probable depression than those whose partners were happy with their pregnancy. Similarly, those who described the support they received from their parents and partners as poor, or fair were more likely to report probable depression than those who received good support (Table 3).

At the community level, perceived neighborhood safety and having support and a safety net within the community were protective against depression symptoms. Pregnant and parenting adolescent girls who perceived their community as safe were less likely to report probable depression in Malawi and Burkina Faso. Safety nets and support within the community were associated with lower odds of probable depression in Burkina Faso but not in Malawi.

## Discussion

Research on depression among pregnant and parenting adolescents is limited in Africa. Such limited focus on depression among this vulnerable group has implications for designing appropriate interventions or advocacy for better access to care. Our study contributes to the evolving literature on the topic by using the widely used socio-ecological model [21, 23, 24] to examine the factors associated with probable depression among PPA girls in Burkina-Faso and Malawi. We found a 14.5% and 18.8% prevalence of probable depression in Malawi and Burkina Faso, respectively. The prevalence of probable depression in the two study settings is consistent with previous studies reporting a prevalence of 13% in Zimbabwe [44] and 16% in South Africa [45]. However, studies among pregnant adolescent girls in Rwanda (48%) [46] and Kenya (33% and 52%) [47, 48] using the Edinburgh Postnatal Depression Screen tool found a far higher prevalence. The varying prevalence of probable depression is due to some differences in the periods of pregnancy and maternity taken into account (many studies focus on the antenatal or postpartum periods) and the use of different screening tools [49].

Factors associated with probable depression in both study settings are broadly similar, but a few differences exist. While age and education were the only individual-level factors found statistically significant in Malawi, only birth status was significantly associated with probable depression in Burkina Faso. Increasing age was associated with high levels of possible depression, while having a secondary education was protective. Our finding on the link between age and depression is consistent with a previous study in Tanzania [50]. The plausible explanation for the association between age and probable depression is that increasing age is linked to adult responsibility [51]. Previous studies have shown the protective benefit of having a higher education level against depression [51, 52]. The protective effect of education against depression in Malawi suggests that girls with some secondary education could be more hopeful for a future career than girls with no education or only primary education. In Malawi, higher education is linked to better future career prospects than no education or having only primary education.

Consistent with previous studies that reported a higher prevalence of depression among pregnant adolescents [46–48], our Burkina Faso result shows that girls with one or more birth were less likely to show symptoms of depression compared to those currently pregnant. This group of girls may have had time to deal with the disappointment of early and unintended pregnancy and devised a coping strategy. Our data shows that some got married and received good support from their partners, which could contribute to helping them overcome the disappointment and fear of early childbearing. Because communities frown at out-of-wedlock pregnancies and exclude such girls, child marriage is often used as a way to restore family honor or avoid public ridicule [53, 54]. Girls who get married may, therefore, be able to overcome the stigma associated with adolescent pregnancies.

At the household and family level, exposure to IPV, parental support, partner support, and paternity denial were associated with probable depression. All four factors were significantly associated with probable depression in Burkina Faso, but only IPV and parental support were significant in Malawi. The finding on the association between IPV and probable depression is consistent with previous studies [55–58]. The plausible explanation is that when adolescent girls become pregnant, often outside wedlock, they voluntarily or involuntarily move out of their parent’s house to live with their partners. Parental rejection makes their partner the only available form of support. If the partner abuses them physically, emotionally, and sexually, they can become hopeless or even suicidal, increasing their risk of depression.

One key finding of this study is that paternity denial is a risk factor for probable depression in PPA girls, as we found in Burkina Faso. Paternity denial has serious negative effects on girls. When a girl becomes pregnant outside wedlock, she is subjected to ridicule and shame, and it gets worse if there is no one accepting responsibility for her pregnancy. She is viewed as promiscuous, lacking honor, and excluded by friends, family, and the community. Denial of paternity means a lack of material provision or child care support from the partner [29, 59]. In addition, denial of paternity sometimes exposes girls to rejection by their own families. The burden of childcare and resource provision rests on them entirely, even though they are unemployed. Paternity denial also represents betrayal, and studies suggest adolescent girls often contemplate abortion or killing their partners [29, 59]. In some of Burkina Faso’s ethnic groups, such as the Mossi, where most of our participants belong, cultural norms prohibit an unmarried pregnant girl from living in the same household as her male family members, especially her father [60]. It is believed that if such girls are allowed to live with their male family members, it could result in deaths or other misfortunes happening to members of the family. For this reason, the girls who become pregnant must voluntarily or involuntarily join the partner responsible for her pregnancy, or alternatively her paternal aunt, who then acts as mediator. It is usually after her delivery that the girl can return to the parental home after rites of forgiveness. Girls dealing with these situations, combined with partner rejection, can lead to extreme distress, sadness and hopeless and losing interest in living.

In contrast, parental and partner support is protective against depression among pregnant and parenting adolescents. This finding is consistent with previous studies [19, 61]. Young mothers need lots of help to navigate their new role as parents. Childcare is tedious and requires human and material resources. Having access to adequate support could help build young mothers' resilience, determination, and self-esteem [62, 63]. Increasing the level of material and non-material support could be an important strategy for addressing poor mental health among pregnant and parenting adolescent girls.

Consistent with previous studies in the global north, our study also shows that community-level factors are associated with probable depression among pregnant and parenting adolescent girls [31, 32]. Unsafe neighborhoods are associated with an increased risk of depression. Fear of violence and actual exposure to violence in one’s community could increase one’s level of stress and anxiety. Neighborhoods with elevated levels of violence and crime are characterized by weak social ties, segregation, and the concentration of low-income earners [64]. However, it is important to interpret our findings with caution given that these are young girls’ perceptions about safety in their community, and two people in the same community may differ in their perceptions.

Poverty has been linked to an elevated risk of depression. As such, the association between unsafe neighborhoods and depression could be bi-directional. We also found that having safety nets and support within the community was associated with lower odds of probable depression. This finding suggests that interventions to address depression among pregnant and parenting adolescents should be multidimensional and draw from community resources to be effective.

## Limitation and strengths

Our use of the socio-ecological model to explain the factors associated with depression is an important strength of this study. Also, our study drew data from pregnant and parenting adolescent girls in two African countries and adds to the body of evolving literature on the topic. However, our study is not without some limitations. Our data is limited to only one main city and environs in the country and is not nationally representative even though it offers important insights into the problem. Also, the evaluation of depressive illness is often carried out within clinical settings by healthcare workers. Because health professionals are much more trusted and can help address these mental health challenges, the information provided by PPA girls in such clinical settings is likely more reliable and accurate. Self-reporting of depression symptoms is subjected to social desirability bias. Since we used enumerators to administer the screening tool, it is possible that our study has underreported the prevalence of depression. Under-reporting of PPA girls who had had an abortion but are not currently pregnant or with a child during the listing stage is likely, given that abortion is highly stigmatized in the study settings.

## Conclusion

This study evaluated the prevalence of probable depression among pregnant and parenting adolescents in Burkina Faso and Malawi. It used the socio-ecological framework to identify factors associated with depressive symptoms. Probable depression symptoms are common among pregnant and parenting adolescents, suggesting the need to regularly screen them for depression during antenatal and postnatal checks. Factors associated with depression among pregnant and parenting girls operate at multiple levels suggesting a need for multilevel interventions that address all areas of vulnerabilities. Also, factors associated with probable depression slightly differ between our two study settings, suggesting a need for context-specific intervention.

## Data Availability

Data will be made available on reasonable request to the corresponding author.

## Abbreviations

EA: Enumeration area
PPA: Pregnant and parenting adolescent
